# Experimental evolution of sperm competitiveness in a mammal

**DOI:** 10.1186/1471-2148-11-19

**Published:** 2011-01-20

**Authors:** Renée C Firman, Leigh W Simmons

**Affiliations:** 1 Centre for Evolutionary Biology (M092), University of Western Australia, 35 Stirling Hwy, Nedlands, 6009, Australia

## Abstract

**Background:**

When females mate with multiple partners, sperm from rival males compete to fertilise the ova. Studies of experimental evolution have proven the selective action of sperm competition on male reproductive traits. However, while reproductive traits may evolve in response to sperm competition, this does not necessarily provide evidence that sperm competitive ability responds to selection. Indeed, a study of *Drosophila *failed to observe divergence in sperm competitive ability of males in lines selected for enhanced sperm offence and defence.

**Results:**

Adopting the naturally polygamous house mouse (*Mus domesticus*) as our vertebrate model, we performed an experimental evolution study and observed genetic divergence in sperm quality; males from the polygamous selection lines produced ejaculates with increased sperm numbers and greater sperm motility compared to males from the monogamous lines. Here, after 12 generations of experimental evolution, we conducted competitive matings between males from lineages evolving under sperm competition and males from lineages subject to relaxed selection. We reduced variation in paternity arising from embryo mortality by genotyping embryos *in utero *at 14 days gestation. Our microsatellite data revealed a significant paternity bias toward males that evolved under the selective regime of sperm competition.

**Conclusion:**

We provide evidence that the sperm competitiveness phenotype can respond to selection, and show that improved sperm quality translates to greater competitive fertilisation success in house mice.

## Background

When females mate with multiple partners, sperm from rival males compete to fertilise the ova [1]. Sperm competition has been shown to influence the evolution of testes size and efficiency [2-5], and sperm form and function [6-11]. Studies of experimental evolution have shown that male reproductive traits can evolve in response to sperm competition [12-17]. However, this does not prove that sperm competitive ability responds to selection or how different genotypes contribute to future generations. The assumption that divergence in sperm number and/or quality translates to competitive ability has been demonstrated in only two cases [15,17], and never before in a vertebrate.

It is now recognised that the sperm competitiveness phenotype is a multifarious, transitional trait that can be influenced by genetic interactions between females and/or rival males. In some species, the female genotype plays a major role in determining the outcome of sperm competition [18-20]. Additionally, it seems that sperm competitive ability may also be contingent on the genotypes of the competing males, and ejaculate-by-ejaculate interactions. Indeed, males have been shown to display non-transitivity in their sperm competitiveness [21,22]. In *Drosophila*, the repeatability of sperm competitiveness declines when ejaculate × ejaculate × female combinations become inconsistent [23], and *in vivo *observations of sperm within the female tract have demonstrated the complexity of these interactions [24]. On top of this, fertilisation and/or paternity success is only a relative predictor of sperm competitive ability dependent upon the stochastic 'background' against which it is measured [25]. Collectively, these factors might constrain sperm competitiveness from responding to selection.

We have generated replicate selection lines of house mice evolving with and without sperm competition. In the eighth generation, we observed genetic divergence in ejaculate quality; males evolving under sperm competition had higher sperm numbers and better sperm motility compared to males with a selection history of monogamy [12]. Here, in the 12^th ^generation we conducted competitive matings using males from our selection lines, and investigated whether the sperm competitiveness phenotype responds to selection by determining whether improved sperm quality translates to superior sperm competitiveness in house mice.

## Results

**Figure 1:**
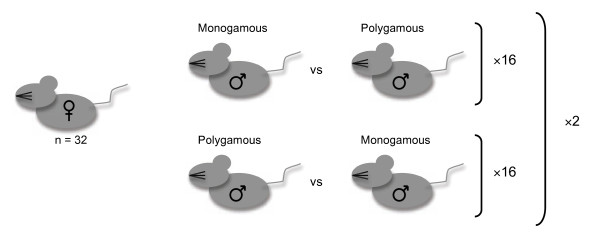
**The experimental design**. Competitive male combinations were generated via a semi-factorial design, creating 32 male combinations. Using all replicate lines the different combinations of monogamous line and polygamous line males were assigned to females with either selection history. To eliminate potential confounding affects due to coevolution within the lines, males and females from the same replicate line did not mate. The entire design was replicated.

**Figure 2:**
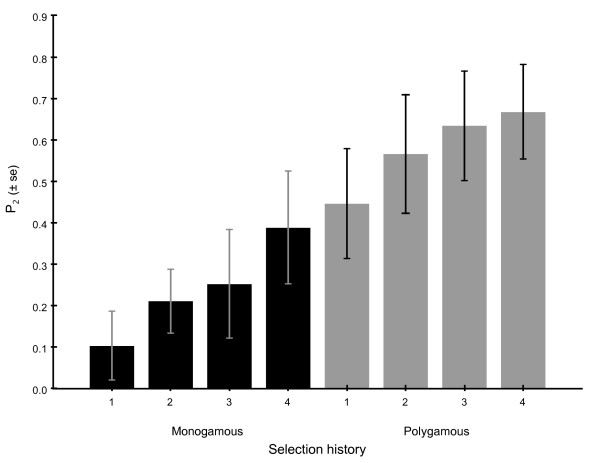
**Sperm competitiveness scores (mean P_2 _± s.e.) of males from the polygamous and monogamous selection lines**.

We used sexually mature male and female house mice from our established selection lines that had been evolving with (polygamous) and without (monogamous) sperm competition for 12 generations, and performed competitive matings between males (Figure 1). A first male to mate advantage (relative to the time of ovulation) is a general pattern of paternity in mammals [26]. For example, in competitive matings of mice the proportion of offspring sired by the second male to mate (P_2_) is close to 0.20 [27]. Here we assess P_2 _for males with a polygamous selection history when competing against males with a monogamous selection history, and vice versa. To quantify female effects on sperm use, the experimental females were taken from both the monogamous and polygamous selection lines. We examined the relative sperm competitiveness of males from the monogamous and polygamous lines by including the proportion of offspring sired by a male when mating in the offensive role of sperm competition (P_2_) in a nested ANOVA. Although 53% of the litters were multiply sired, our microsatellite data revealed a significant paternity bias toward males from the polygamous selection lines. Thus, there was a significant effect of male selection history on the proportion of embryos sired by the second male to mate (P_2_) (Figure 2), but no effect of female selection history (Table 1). The average proportion of offspring sired by males from the polygamous lines when they competed against males from the monogamous lines (0.58 ± 0.06) was significantly higher than the proportion of offspring sired by monogamous males when competing against males from polygamous lines (0.24 ± 0.06) (Figure 2). In both the defensive (i.e. first to mate) and offensive (i.e. second male to mate) roles, males from the polygamous lines gained exclusive paternity of 33% of the litters, while males from the monogamous lines gained exclusive paternity of just 14% of the litters.

**Table 1:** ANOVA of the proportion of offspring sired by a male when mating in the disfavoured role of sperm competition (P_2_).

Effect	SS	df	MS	*F*	*P*
Male selection history	12.51	1, 6	12.51	32.88	**0.008**
Male line[selection history]	2.68	6, 49	0.45	0.50	0.804
Female selection history	3.22	1, 6	3.22	5.00	0.070
Female line[selection history]	3.91	6, 49	0.65	0.73	0.625
					

## Discussion

We conducted competitive matings between male house mice from polygamous and monogamous selection lines, and found that males evolving with sperm competition (polygamy) had a significant paternity bias over males evolving without sperm competition (monogamy). We thus provide evidence that the sperm competitiveness phenotype can respond to selection, and show that improved sperm quality translates to greater competitive fertilisation success in a mammal.

Male mice have been shown to adjust their ejaculate expenditure according to the perceived risk of sperm competition [28]. Under the established mating regimes of the selection experiment, it is possible that males from the polygamous lines became more sensitive to cues that are indicative of female mating history, which thereby influenced their copulatory behaviour and competitive ability. In a previous experiment we conducted remote observations of copulatory behaviour in a monandrous and polyandrous context [27]. Mating behaviour did not differ between the two treatments, suggesting that males do not adjust their copulatory behaviour according to the number of previous partners a female had mated with [27]. Following eight generations of selection, we assessed the sperm quality of males from the monogamous and polygamous selection lines [12]. Our analyses revealed genetic divergence between the two selective regimes, and provided evidence that sperm traits had evolved in response to sperm competition. We found that males from the polygamous lines had ejaculates with greater numbers of sperm and better sperm motility compared to males from the monogamous lines [12]. Given that the mean sperm quality scores and the mean P_2 _values were derived from different generations (eight and 12 respectively) we are unable to test directly for a correlation between sperm quality and sperm competitiveness among our selection lines. However, it is striking that the mean sperm quality scores from generation eight [12] follow the rank order of mean P_2 _values found here across the replicate selection lines (Figure 2). Indeed, we have shown that the males from the polygamous lines have evolved ejaculates with greater numbers of sperm and increased sperm motility [12]. Improved sperm quality in the polygamous lines may have been driven by directional selection on standing genetic variation, and/or via the removal of deleterious mutations that suppress sperm quality [12]. Regardless of the precise mechanism for the divergence, it would appear that the polygamous males outcompeted their rivals by reaching the ova first and/or by penetrating the ova first, and thereby gaining greater numbers of fertilisations. Indeed, competent sperm motility is an important determinant of fertilisation success in vertebrates [29,30].

It is also possible that a paternity bias toward the polygamous males could have been generated (or magnified) via preferential implantation of ova fertilised by males from the polygamous line. It has been proposed that females can benefit from polyandry when genetically superior males are successful in sperm competition and transfer good genes to their offspring [31,32]. Studies of invertebrates have shown that additive genetic contributions, such as intrinsic male effects, can contribute to offspring fitness [33,34]. Thus, males from our polygamous lines may have evolved an intrinsic trait that improved zygote quality and ensured higher rates of implantation. Males from the polygamous lines sired a higher proportion of offspring compared to males from the monogamous lines. Additional experimentation is required to further assess the paternity success of the polygamous males, and to separate potential effects of preferential zygote implantation from competitive ejaculate quality.

In studies of postcopulatory sexual selection, paternity success at birth has routinely been applied as a measure of male fertilisation success and sperm competitiveness. These studies have provided convincing support for the evolution and maintenance of polyandry based on the good genes [31,32], and compatible genes hypotheses [35]. However, inequalities between paternity success at birth and fertilisation success may have significant consequences for studies assessing sperm competitive ability and postcopulatory paternity biasing mechanisms [36]. Unfortunately, there are methodological limitations to achieving this in internally fertilising species. Here, we reduced variation in paternity arising from embryo mortality by genotyping embryos *in utero *at 14 days gestation. Thus, our study provides a more accurate estimate of fertilisation success and sperm competitiveness than has previously been used in studies of mammals.

## Conclusion

In conclusion, we have shown that polygamy influences the evolvability of the sperm competitiveness phenotype in house mice. Previously, following eight generations of experimental evolution, we found that males with a selection history of polygamy evolved ejaculates with more sperm and better sperm motility compared to males with a selection history of monogamy [12]. Here, by conducting competitive matings between males with selection histories of monogamy and polygamy, we provide the first demonstration that polygamy selects for superior sperm competitiveness in a vertebrate.

## Methods

### Experimental animals and selection lines

All the experimental procedures outlined below were assessed and approved by an animal ethics committee at the University of Western Australia (07/100/607). As we have described previously [12], we established eight selection lines by recruiting animals from 60 litters generated by a colony of wild-type mice maintained at the Animal Resources Centre (Murdoch, Western Australia). We founded replicate lineages that were mated monogamously maintained via the middle-class neighbourhood design; males and females mated monogamously and contributed two offspring (one male, one female) to each subsequent generation [37]. Thus, we relaxed selection on juvenile fitness by providing food *ad libitum *and separate housing to gestating and nursing females, and eliminated almost all selection on adult fitness by guaranteeing that every male and female pair contributed one son and one daughter to the next generation. Although greatly relaxed, selection could not be completely eliminated because rarely a pair did not mate or produce offspring. We also established replicate polygamous lines in which adult females had equal fitness (two offspring), and adult males had equal mating success but not equal fertilisation success due to the postcopulatory process of sperm competition. Thus, selection did not operate in the monogamous lines (M), and precopulatory sexual selection did not operate in the polygamous lines (P). Sperm competition (postcopulatory sexual selection on males) operated only in the polygamous lines.

Four monogamous lines were each established with 18 males and 18 females. Subsequently, 18 males and 18 females contributed to each generation. That is, every fecund pair contributed a son and daughter to the next generation. Four polygamous lines were established with 18 females and 18 males, but potentially < 18 sires. In the P-lines, the same three males mated with the same three females. Thus, males in the P-lines competed for fertilisations, and the number of males that contributed to successive generations was determined by the relative paternity success of each male. As with the monogamous lines, one male and one female were selected at random from each polygamous line litter and used to produce the next generation. In the case of single sex litters, two males or two females were mated to produce the subsequent generation. Nests were checked for pups beginning 19 days after mating. Litters were weaned at three weeks of age, and separated at four weeks of age. Animals were deemed sexually mature at eight weeks of age.

### Experimental design of competitive matings

Here, we used sexually mature mice from generation 12 of our selection lines. Competitive male combinations were generated via a semi-factorial design whereby males from the four replicate monogamous lines competed against males from the four replicate polygamous lines, and vice versa (4 M-lines × 4 P-lines), thus creating 32 male combinations (16 M × P; 16 P × M) (Figure 1). Using all replicate lines, we randomly assigned different combinations of M-line and P-line males to females with either selection history. However, to eliminate potential confounding affects due to coevolution within lines, males and females from the same replicate line did not mate. The entire design was replicated so that the total number of experimental matings equalled 64. Male body size did not differ between the two selection regimes (*F*_1, 6 _= 0.091, *P *= 0.773), or replicate lines nested within selection history (*F*_6, 56 _= 1.615, *P *= 0.160) (whole model: *F*_7, 56 _= 1.405, *P *= 0.221; mean body weight: P = 21.26 ± 0.40 g, M = 21.46 ± 0.37 g). Similarly, female size did not differ (selection treatments: *F*_1, 6 _= 2.634, *P *= 0.156; replicate selection lines: *F*_6, 56 _= 1.053, *P *= 0.402; whole model: *F*_7, 56 _= 1.299, *P *= 0.268; mean body weight: P = 21.48 ± 0.46 g, M = 22.43 ± 0.33 g).

### Experimental matings

The animals used in this experiment were from the 12^th ^generation of the selection lines. Matings were conducted during the dark phase of a 10:14 hour reversed light:dark cycle, under a red light. Females were inspected every 2 hours for oestrus condition [38]. When females were in oestrus they were allocated a male and checked half hourly for the presence of a mating plug. Once a mating plug was observed it was removed by gently pressing the female against the side of the handling bin and dislodging it with a blunt probe. The female was then paired with a second male and again checked every half hour for the presence of a plug. Once the second mating was achieved the female was placed in a clean box with shredded newspaper for nesting. From a previous experiment we know that plug removal does not affect fertility in mice [12]. Mating sessions typically began at hour 7 of the dark phase, and lasted between 1 and 5.5 hours. The time taken to achieve ejaculation in the disfavoured role of sperm competition did not differ between M-line (mean = 2.0 ± 0.2 hours) and P-line males (mean = 1.7 ± 0.1 hours) (ANOVA: *F*_1, 62 _= 2.48, *P *= 0.121).

### Paternity analysis

Fourteen days after mating, females were sacrificed (lethal injection) and dissected, and the embryos were removed from the reproductive tract. The number of embryos at 14 days gestation did not differ between females from the monogamous (mean = 7.91 ± 0.29) and polygamous lines (mean = 7.59 ± 0.36) (*F *_1, 6 _= 0.22, *P *= 0.655); although there was a significant effect of replicate line nested within selection history (*F *_6, 56 _= 2.35, *P *= 0.042) (whole model: *F *_7, 56 _= 2.10, *P *= 0.059). DNA was extracted from embryonic and parental (ear) tissue using the EDNA HISPEX extraction kit (Fisher Biotec, Subiaco, Western Australia). Paternity was unambiguously assigned to 411 embryos by screening four microsatellite loci (D4Mit1, D10Mit14, D13Mit 1, D18Mit17); it was necessary to screen three additional loci to assign paternity to 85 embryos (D6Mit138, D11Mit4, D14Mit132) [39,40]. Labeled primers were obtained from GeneWorks (Hindmarsh, South Australia) (FAM) and Applied Biosystems (Foster City, California) (NED, PET, VIC) and unlabeled primers from GeneWorks. Primers were multiplexed in 10 μl reactions in a PTC-0200 DNA engine (GeneWorks). Reactions contained 5 or 6 μl of a multiplex kit (Qiagen, Doncaster, Victoria), 0.25 μM of forward labeled primer, 0.25 μM of reverse primer, and ~ 200 ng of template DNA. The thermocycling profile for all loci was: 5 min denature at 95°C, 50 cycles of 90°C for 20 s, 55°C for 20 s, and 72°C for 30 s, followed by 72°C for 3 min. PCR products (1.5 μl) were run on a ABI3730 Sequencer, sized using Genescan-500 LIZ size standard and genotyped using Genemapper software (ver. 3.0) (Applied Biosystems). Paternity was assigned by manual exclusion.

Our microsatellite data revealed that individuals from the two selection treatments did not differ in the level of heterozygosity (*F*_1, 6 _= 0.590, *P *= 0.472; mean *H*_O_: P = 0.49 ± 0.09, M = 0.56 ± 0.04), or average inbreeding coefficient (*F*_1, 6 _= 0.61, *P *= 0.465; mean *F*_IS_: P = 0.14 ± 0.07, M = 0.09 ± 0.03).

### Data analysis

Our independent unit of replication was the number of lines for each selection treatment (n = 8). To obtain the appropriate error degrees of freedom in the analyses we conducted nested ANOVAs with replicate line nested within selection history as a random factor. All means are presented ± 1 se.

## Authors' contributions

RCF and LWS developed the concept of the experiment. RCF reared the selection lines, and performed the experimental matings, sperm assays and paternity analysis. Both authors read and approved the final manuscript.
